# NeuralVisionNet: a probabilistic neural process model for continuous visual anticipation

**DOI:** 10.3389/fncom.2026.1781080

**Published:** 2026-03-24

**Authors:** Han He, Ruinan Chen, Yixiang Wang, Xia Chen

**Affiliations:** 1 Faculty of Data Science, City University of Macau, Taipa, Macao SAR, China; 2 University of Wisconsin – Milwaukee, Milwaukee, WI, United States

**Keywords:** attentive neural processes, continuous visual anticipation, neural computing, neuroscience, probabilistic neural process

## Abstract

The ability to anticipate future events continuously is a hallmark of biological vision, yet standard deep learning models often struggle with long-term coherence due to the rigid discretization of time. In this paper, we propose NeuralVisionNet, a probabilistic framework that models visual anticipation as a continuous generative process, drawing inspiration from the predictive coding mechanisms of the hippocampal-entorhinal circuit. Our architecture synergizes hierarchical Video Swin Transformers with Attentive Neural Processes, employing a novel grid-like coding scheme to represent spatiotemporal dynamics as a continuous function rather than a fixed sequence of frames. Furthermore, we introduce a variational global latent variable to encode the “event gist,” ensuring semantic consistency over extended horizons. Extensive evaluations on KTH, Human 3.6M, and UCF 101 benchmarks demonstrate that NeuralVisionNet significantly outperforms state-of-the-art stochastic baselines in perceptual quality (FVD) and structural fidelity (SSIM), offering a robust computational proof-of-concept for continuous, bio-inspired visual forecasting.

## 1 Introduction

The capacity to anticipate future sensory states is a fundamental computational principle of the brain, enabling biological agents to compensate for neural transmission delays and navigate dynamic environments. Under the framework of predictive coding, cortical circuits are hypothesized to maintain an internal generative model that constantly predicts incoming sensory signals, minimizing the variational free energy between expectations and reality (Friston, 2010; Millidge et al., 2022). While this biological imperative has inspired a wave of deep learning models for video prediction, the landscape has recently shifted toward large-scale “World Models.” Notable recent advancements, such as the video generation model Sora (Bardes et al., 2024) and Lumiere (Bar-Tal et al., 2024), have demonstrated an unprecedented ability to simulate physical dynamics. However, these massive systems often function as “black boxes,” relying on computationally intensive diffusion processes that diverge significantly from the energy-efficient, sparse predictive mechanisms observed in biological circuits. Furthermore, while Joint-Embedding Predictive Architectures (V-JEPA) have recently been proposed to learn semantic representations without pixel-level generation (Bardes et al., 2024), they often forgo the reconstruction of detailed sensory reality, which is essential for immediate sensorimotor control.

To bridge the dichotomy between discrete deep learning and continuous biological processes, Implicit Neural Representations (INRs) and Neural Processes (NPs) have emerged as powerful tools. INRs parameterize data as continuous functions, offering resolution-independent modeling. Concurrently, Neural Processes combine the functional flexibility of neural networks with the probabilistic uncertainty estimation of Gaussian Processes. However, applying these to high-dimensional video remains challenging. While recent works have begun to explore 4D scene representations using Gaussian Splatting (Wu et al., 2024), integrating these with the hierarchical, semantic reasoning of the visual cortex remains an open problem. Specifically, modeling complex video dynamics requires an architecture that can simultaneously handle local visual details and global spatiotemporal contexts, mimicking the interactions between the visual cortex and the medial temporal lobe.

In this paper, we introduce NeuralVisionNet, a probabilistic framework for continuous visual anticipation that draws direct inspiration from the brain's predictive mechanisms. We propose a hierarchical architecture that couples a Video Swin Transformer (Liu et al., 2022) with an Attentive Neural Process. Crucially, we implement a grid-like coding scheme to serve as a continuous spatiotemporal coordinate system, analogous to the biological grid cell code. This allows our model to generate predictions at arbitrary continuous time steps, simulating the fluidity of biological motion perception. Furthermore, by introducing a variational global latent variable to encode the “event gist,” our model ensures semantic consistency over long horizons. We demonstrate that NeuralVisionNet not only competes with recent state-of-the-art baselines but also exhibits neuro-computational properties aligned with the latest theories of episodic memory and predictive coding.

## 2 Related work

### 2.1 Neuro-computational mechanisms of episodic memory and prediction

Contemporary frameworks in computational neuroscience, most notably the Free Energy Principle, posit that the brain functions as a predictive inference engine, continuously minimizing variational free energy by aligning top-down generative models with bottom-up sensory inputs (Millidge et al., 2022). Central to this anticipatory capability is the hippocampal-entorhinal circuit, which orchestrates episodic memory by binding specific sensory experiences to a flexible, abstract spatiotemporal framework. Specifically, grid cells in the medial entorhinal cortex are hypothesized to provide a low-dimensional, metric coordinate system—a “cognitive map”—that supports not only spatial navigation but also the “mental time travel” required to simulate future event trajectories (Moser et al., 2008; Bellmund et al., 2018). Recent advances in “NeuroAI” have begun to operationalize these biological principles; for instance, the Tolman-Eichenbaum Machine (TEM) demonstrates how separating sensory representations from structural coordinates allows for robust generalization and zero-shot inference (Whittington et al., 2020). However, a critical distinction emphasized in recent reviews is the inherent continuity of biological codes compared to artificial implementations; while the brain leverages continuous attractor manifolds to represent fluid dynamics, most computational models rely on discretized state spaces, highlighting the need for probabilistic architectures that can faithfully model the continuous evolution of spatiotemporal “gists” (Zador et al., 2023).

### 2.2 Deep generative models for spatiotemporal prediction

The paradigm of video anticipation has rapidly shifted from task-specific architectures to large-scale foundation models that generalize across diverse visual domains. While earlier efforts relied on localized operations (Shi et al., 2015), the current state-of-the-art is dominated by massive Transformer-based architectures, such as VideoMAE V2 and InternVideo, which leverage masked autoencoding to capture long-range spatiotemporal dependencies with unprecedented semantic depth (Tong et al., 2022; Wang et al., 2023). Most recently, this trajectory has culminated in the development of “World Simulators” like Sora (Brooks et al., 2024), Genie (Bruce et al., 2024), and Lumiere (Bar-Tal et al., 2024). These systems, often built upon latent diffusion or autoregressive tokenization, exhibit a remarkable ability to model complex physical interactions and maintain structural coherence over extended durations. However, a fundamental theoretical limitation persists: these approaches predominantly rely on discrete representations—quantizing continuous time and space into fixed visual tokens or pixel grids (Blattmann et al., 2023). This discretization introduces an “ontological gap,” as it fails to capture the continuous nature of motion signals or the resolution-independent properties of biological vision.

### 2.3 Positioning neuralvisionnet against three lines of video forecasting

Recent progress in video forecasting can be organized into three lines that are closely related to our design. First, implicit neural video representations treat a video as a continuous function of coordinates or indices, enabling resolution independent synthesis and interpolation; representative examples include Fourier feature based coordinate mappings and neural video representations that regress frames from time indices, as well as recent dynamic scene function parameterizations such as 4D Gaussian based representations (Chen et al., 2021; Wu et al., 2024). These methods excel at compact continuous representations and reconstruction, but they are typically not formulated as conditional stochastic forecasting mechanisms that explicitly infer a distribution over plausible futures from a context set. Second, neural processes for spatiotemporal forecasting provide a probabilistic function learning view that maps an arbitrary sized context set to a predictive distribution over targets, which is attractive for uncertainty aware forecasting; attentive variants and spatiotemporal adaptations have shown how to scale this idea beyond simple regression settings (Kim et al., 2019; Wu et al., 2024). Our approach follows this probabilistic function learning perspective but instantiates it with a hierarchical video encoder and a global latent that is shared across the predicted horizon, so the model samples one coherent high level intent rather than resampling per step. Third, transformer based video predictors and modern world models dominate large scale training, including efficient transformer predictors, masked video modeling backbones, diffusion based video prediction, and recent text to video or token based world simulators (Voleti et al., 2022; Ye and Bilodeau, 2022; Bar-Tal et al., 2024; Brooks et al., 2024). While these approaches achieve strong realism and long range modeling, they largely operate on discretized frames or tokens, making time quantization a core design choice; in contrast, NeuralVisionNet targets continuous time querying with a probabilistic latent memory, aiming to improve temporal coherence under uncertainty and enabling interpolation at arbitrary time steps without changing the discrete rollout length.

## 3 Proposed method

### 3.1 The overall network

**Figure 1:**
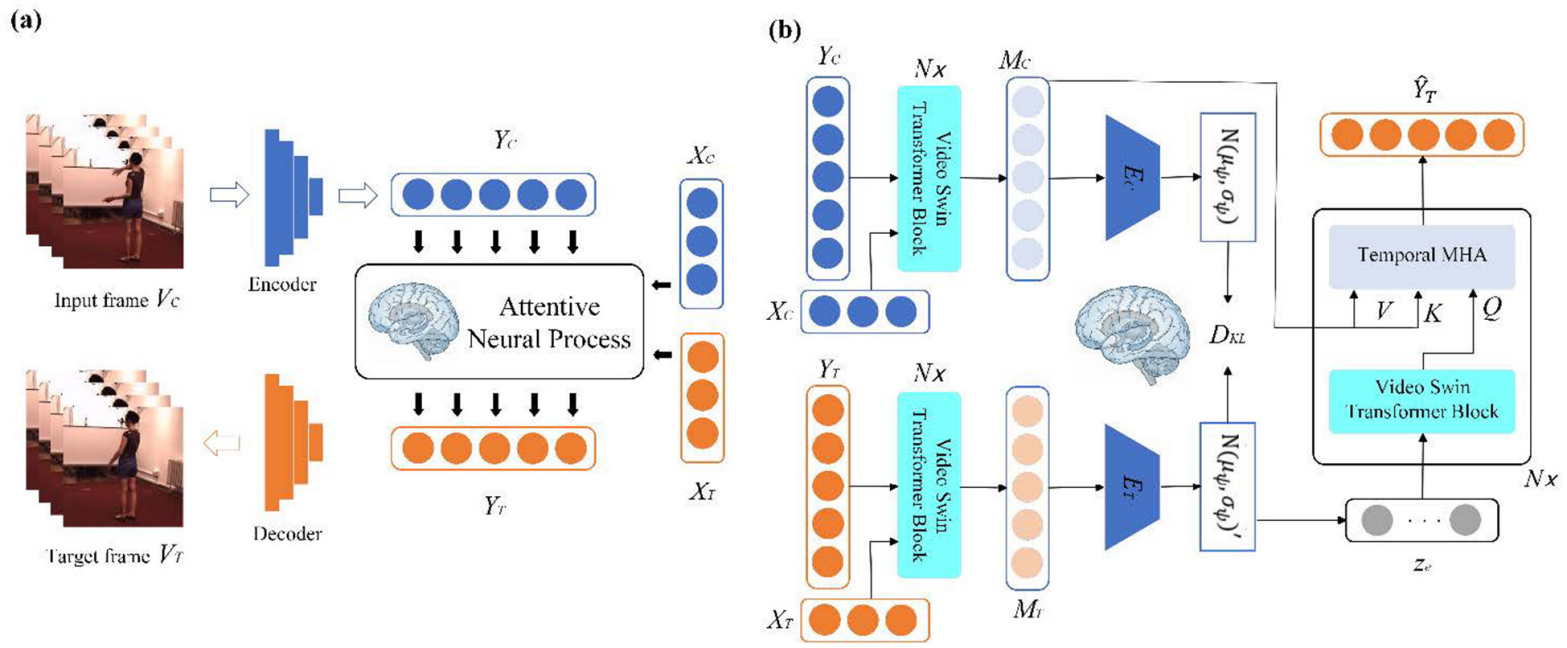
Overview of the NeuralVisionNet architecture. **(a)** The overall framework. High-dimensional video frames (*V*) are compressed into abstract features (*Y*) by the Sensory Encoder, while spatiotemporal dynamics are modeled by the Attentive Neural Process (ANP). **(b)** The internal mechanism of the ANP. It employs Video Swin Transformers to encode context/target streams into a probabilistic global latent variable z_*e*_. Future predictions $\hat{Y}$_*T*_are generated by a Transformer Decoder that queries z_*e*_ using target coordinates (X_*T*_) via temporal multi-head attention, optimized using a variational ELBO objective involving D_*KL*_.

As illustrated in Figure 1, NeuralVisionNet is a probabilistic framework designed to emulate the hierarchical predictive architecture of the mammalian visual cortex, treating video anticipation as a continuous generative process. The system pipeline operates in three functionally distinct stages corresponding to sensation, cognitive abstraction, and generative recall. First, high-dimensional raw video streams are compressed into compact, abstract neural representations via an attention-augmented Sensory Encoder, which effectively decouples static visual features from temporal dynamics. Second, to model the continuous flow of motion, these features are bound with spatiotemporal coordinates using a neuromorphic grid-like coding scheme and processed by an ANP. Crucially, the ANP acts as an episodic memory module, utilizing Video Swin Transformers to distill past context into a stochastic global latent variable-the “event gist-which captures the high-level semantic intent of the sequence. Finally, a Generative Decoder reconstructs future frames by querying this latent memory trace with specific continuous time coordinates, enabling the system to perform “mental time travel” and generate coherent predictions at arbitrary temporal resolutions.

### 3.2 Sensory encoding with attentional modulation

To emulate the hierarchical processing of the biological visual system, we employ a frame autoencoder to transform high-dimensional raw sensory inputs (video frames) into compact, abstract neural representations. This module serves as the sensory front-end, decoupling the learning of static visual features from the modeling of temporal dynamics.

Specifically, the encoder architecture is adapted from the Pix2Pix framework1, designed to map pixel-space stimuli into a latent feature space *Y*∈ℝ^*H*×*W*×*D*^. Crucially, to overcome the limitation of local receptive fields in standard convolutional layers, we integrate non-local attention mechanisms 2 (depicted as orange layers in Figure 1a). From a computational neuroscience perspective, this mechanism mimics the long-range horizontal connections or top-down attentional modulation seen in the visual cortex, allowing neurons to integrate global contextual information beyond their immediate receptive fields. This ensures that the encoded features *Y*_*C*_ and *Y*_*T*_ capture globally coherent semantic structures essential for the subsequent associative memory tasks.

The sensory encoder and decoder are trained via a pixel-level reconstruction objective using an *l*_1_ loss. Let *I* ∈ ℝ^*H*×*W*×*C*^ denote an input RGB frame and Î denote its reconstruction produced by the decoder. The reconstruction loss is defined as:


$$\begin{array}{l} L_{rec}=||I-Î{||}_{1}=\frac{1}{HWC}\underset{h,w,c}{\sum }|I_{h,w,c}-\;{Î}_{h,w,c}| \end{array}$$

Where ||▪||_1_ denotes the element-wise *l*_1_ norm (mean absolute error) over all pixels.

### 3.3 Continuous spatiotemporal representation via grid-like coding

Unlike traditional video prediction models that treat time as a discrete sequence of frames, biological vision perceives motion as a continuous flow. To endow our model with this capability, we employ Implicit Neural Representations (INRs) parameterized by a Fourier Feature Network (FFN). From a neuromorphic perspective, this module functions analogously to the grid cells and time cells found in the entorhinal cortex, which provide a metric coordinate system for spatial navigation and episodic memory.

Specifically, for any spatiotemporal coordinate *c* (e.g., a 3D coordinate including spatial position and time), the Fourier Feature Network (FFN) maps this low-dimensional coordinate into a high-dimensional position encoding vector *x*_*c*_. This encoding enables the model to represent high-frequency spatial and temporal variations and provides explicit spatiotemporal cues for the subsequent attention-based predictor. This process mimics the spatially periodic firing patterns of grid cells, allowing the model to encode high-frequency spatial details that are often lost in standard coordinate inputs. The encoding is formulated as:


$$\begin{array}{l} x_{c}={\left[\cos\left(2\pi Bc\right),sin\left(2\pi Bc\right)\right]}^{T} \end{array}$$

where B is a learnable projection matrix that controls the frequency bands of the Fourier features.

This continuous coordinate encoding serves two critical neuro-computational functions:

1 Spatiotemporal Binding: Since the subsequent predictor (see Section 3.4) relies on attention mechanisms which are inherently permutation-invariant (similar to the associative nature of hippocampal CA3), these embeddings are essential for binding specific visual features to their precise locations in space and time.

2 Mental Simulation and Interpolation: By defining the visual signal as a continuous function over coordinates rather than a fixed grid of pixels, our model can generalize to unseen temporal coordinates. This allows the system to perform “mental time travel-generating predictions at arbitrary time steps *i* (even fractional ones)-effectively simulating continuous motion dynamics from discrete memory snapshots.

In our Attentive Neural Process formulation, the coordinate encodings are used as the input locations. Concretely, *X*_*C*_ and *X*_*T*_ in Section 3.4 correspond to the collection of FFN outputs {*x*_*c*_} for context coordinates and the FFN output for target co ordinates, respectively. These position encodings provide spatiotemporal cues for attention and are used to condition the predictive distribution of the target visual representations.

### 3.4 Attentive neural process

#### 3.4.1 Modeling event coherence with global latent variables

Human episodic memory is not merely a collection of independent snapshots but a coherent narrative governed by an underlying “gist” or intent. To mimic this neurocognitive mechanism, we design our predictor as an Attentive Neural Process (ANP) instantiated with a Video Swin Transformer Block. While standard Neural Processes often treat data points as independent given the context, this assumption fails to capture the temporal continuity of visual motion.

In biological terms, when the brain predicts a future sequence, it does not re-sample the motion uncertainty at every millisecond (which would lead to jittery, incoherent hallucinations). Instead, it samples a single, high-level “event concept” that guides the entire prediction. We model this by introducing a global latent variable *z*_*e*_ (the “event variable”), which represents the stable, high-level intent or semantic gist of the video sequence.

Mathematically, this follows the Free Energy Principle, where the brain minimizes the variational free energy to align its internal model with sensory reality. We formulate the generative process of the target future representations *Y*_*T*_ as:


$$\begin{array}{l} p_{\theta }\left(Y_{T}|X_{C},Y_{C},X_{T}\right)=\int p_{\theta }\left(Y_{T}|X_{C},z_{e},r_{C}\right)p_{\theta }\left({z_{e}|X}_{C},Y_{c}\right)dz_{e} \end{array}$$

where *p*_θ_(*Y*_*T*_|*X*_*C*_, *Y*_*C*_, *X*_*T*_) serves as the context-conditioned prior belief about the event gist formed from past context memories, and the conditional likelihood *p*_θ_(*Y*_*T*_|*X*_*C*_, *Y*_*C*_, *X*_*T*_, *z*_*e*_)explicitly depends on the sampled event variable *z*_*e*_ to generate coherent future predictions.

We optimize the model by maximizing the Evidence Lower Bound (ELBO), effectively minimizing the surprise of the prediction:


$$\begin{array}{r} L_{ELBO}={𝔼}_{q_{\phi }\left({z_{e}|X}_{C},Y_{C},X_{T},Y_{T}\right)}\left[\log p_{\theta }\left(Y_{T}|X_{T},z_{e},r_{C}\right)\right]-\beta D_{KL}\\ \left(q_{\phi }\left(▪\right)||p_{\theta }\left(▪\right)\right) \end{array}$$

Here, the first term is the reconstruction accuracy (prediction error), and the second term is the complexity cost (KL divergence). The KL term is particularly crucial from a neuroscience perspective: it constrains the “posterior” belief (formed after seeing the future, i.e., conditioned on (*X*_*T*_, *Y*_*T*_) during training) to remain consistent with the “prior” expectation (formed only from context), enforcing a consistent mental model across time. At inference time, since *Y*_*T*_ is unavailable, we sample *z*_*e*_ from the prior *p*_θ_(_*z*_*e*_|*XC*_, *Y*_*C*_).

We model the likelihood *p* as a Laplacian distribution with a constant scale, implying that the brain performs deterministic execution given a specific intent *z*_*e*_. Thus, maximizing likelihood simplifies to minimizing an *L*_1_ loss in the latent space. To further ground the abstract features in sensory reality and correct for manifold curvature, we also impose a pixel-level reconstruction loss via the frozen frame decoder. The final objective is:


$$\begin{array}{l} L_{total}=r|V_{T}-{\hat{V}}_{T}|+|Y_{T}-\hat{Y_{T}}|+\beta D_{KL} \end{array}$$

This multi-level loss reflects the hierarchical error minimization observed in the predictive coding cortex.

#### 3.4.2 Neural architecture for episodic encoding and generation

The architecture, illustrated in Figure 1b, functionally replicates the interplay between memory encoding and generative recall pathways. It consists of three primary modules: a Context Event Encoder (simulating memory consolidation), a Target Event Encoder (providing training supervision), and a Transformer Decoder (simulating future generation).

1 Event Encoders as Abstraction Modules: The Context Event Encoder *T*_*E*_ processes the raw memory traces (*X*_*C*_, *Y*_*C*_) to extract the compact probabilistic representation of the event *z*_*e*_. We utilize the Video Swin block as the backbone due to its spatiotemporal attention mechanism, which aligns with the brain's ability to attend to salient features across space and time. The encoded features *M*_*C*_ are averaged over the temporal dimension-an operation mimicking the temporal compression of episodic memories in the hippocampus-before being mapped to the parameters μ_ψ_, σ_ψ_ of the Gaussian distribution for *z*_*e*_.

2 Generative Recall via Transformer Decoder: The Transformer Decoder *T*_*D*_ acts as the generative engine. It takes the specific spatiotemporal coordinates *X*_*T*_ (where and when to predict) and the global event context *z*_*e*_ (what happens) as inputs. Through cross-attention mechanisms, the decoder “queries” the relevant context memories *M*_*C*_ driven by the coordinate cues *X*_*T*_. This design reflects the constructive nature of memory: the brain does not simply replay a recording but actively reconstructs the future by querying past associations based on current spatial and temporal cues.

During training, the model learns to align the prior distribution (from Context) with the posterior distribution (from Target) via the KL divergence loss. During inference (test time), since the future is unknown, the “event gist” *z*_*e*_ is sampled directly from the learned prior (μ_ψ_, σ_ψ_), allowing the model to hallucinate a coherent future sequence that is semantically consistent with the observed past.

#### 3.4.3 Time coordinate parameterization

We represent the temporal input as a real-valued scalar (float32) and feed it to the Fourier Feature Network together with the spatial coordinates. For each clip, we first assign an integer frame index *i* to every frame. During training, the context window uses indices *i* ∈ [0, *C*−1] and the target window uses indices *i* ∈ [*C, C*+*H*_*train*_−1]. To ensure a consistent temporal scale, we normalize time by a fixed denominator *D* = *C*+*H*_*train*_−1 and set *t* = *i*/*D*. At inference time, we keep the same normalization and query future frames using *t* = *i*/*D* for *i* ∈ [*C, C*+*H*_*test*_−1], which naturally allows *t*>1 and thus supports extrapolation beyond the training horizon. This design keeps the temporal coordinate continuous, while integer indices correspond to samples on the continuous time axis; fractional-time queries can be obtained by directly using non-integer *i* in the same normalization.

## 4 Experiments

### 4.1 Datasets

To validate the robustness and generalization capability of NeuralVisionNet across varying degrees of complexity, we conducted comprehensive evaluations on three diverse real-world video benchmarks: KTH (Schuldt et al., 2004), Human 3.6M (Ionescu et al., 2011), and UCF101 (Soomro et al., 2012). These datasets range from controlled environments to unconstrained dynamic scenes. For all experiments, video frames were resized to a spatial resolution of 64 × 64 to ensure consistency with established baseline comparisons in the literature.

Evaluation Protocol. Following standard protocols for stochastic video prediction, we assessed quantitative performance using Peak Signal-to-Noise Ratio (PSNR), Structural Similarity Index (SSIM), and Fréchet Video Distance (FVD) (Unterthiner et al., 2019). Acknowledging the inherent uncertainty of future events, we adopted the “best-of-N” evaluation strategy common to probabilistic frameworks. Specifically, for each test sequence, we sampled 100 distinct future trajectories (N=100) from the model's learned latent prior. We report the best PSNR and SSIM scores among these samples to measure the model's capacity to cover the ground-truth future mode. Conversely, we report the average FVD score across all samples to evaluate the overall perceptual quality and temporal coherence of the generated distribution. Clarified in the evaluation protocol section that all baselines are evaluated using the same sampling budget and the same reporting rules.

### 4.2 Experimental setup

Following the established baseline configuration (Voleti et al., 2022), we utilize the first 10 frames of each video sequence as the context window to condition the model's predictions. To rigorously evaluate the system's capacity for long-term anticipation and generalization, we adopt distinct horizons for optimization and inference: while the model is trained to reconstruct the immediate next 10 frames to learn local motion dynamics, it is tasked with extrapolating the subsequent 30 frames during the testing phase. Across all datasets and experimental scenarios, quantitative performance is comprehensively assessed using three standard metrics: PSNR for pixel-level fidelity, SSIM for perceptual quality, and FVD for measuring the realism and temporal coherence of the generated distributions.

### 4.3 Implementation details

All experiments were conducted on a single workstation with one NVIDIA GeForce RTX 4090 GPU (24 GB VRAM), an Intel Core i9-13,900K CPU, 64 GB system memory, and a 2 TB NVMe SSD. The software environment used Ubuntu 22.04 LTS, Python 3.10, PyTorch 2.1, and CUDA 12.1 with cuDNN enabled. For training, we used the AdamW optimizer with an initial learning rate of 1e-4 and weight decay of 1e-4. Unless otherwise specified, the batch size was set to 16 (adjusted when limited by GPU memory), and models were trained for 100 epochs. We adopted a cosine learning rate schedule with a linear warm-up of 5 epochs.

### 4.4 Ablation experiments

To examine the contribution of the global event representation, we construct an ablation baseline NeuralVisionNet (w/o event gist) by removing the global “event gist” variable from the original framework. We discard the context and target event distributions and train a deterministic conditional predictor that maps target coordinates and context memory tokens to target latent representations via cross attention. The training objective contains only the reconstruction losses in latent space and pixel space, without the KL divergence term.

### 4.5 Results and analysis

**Figure 2:**
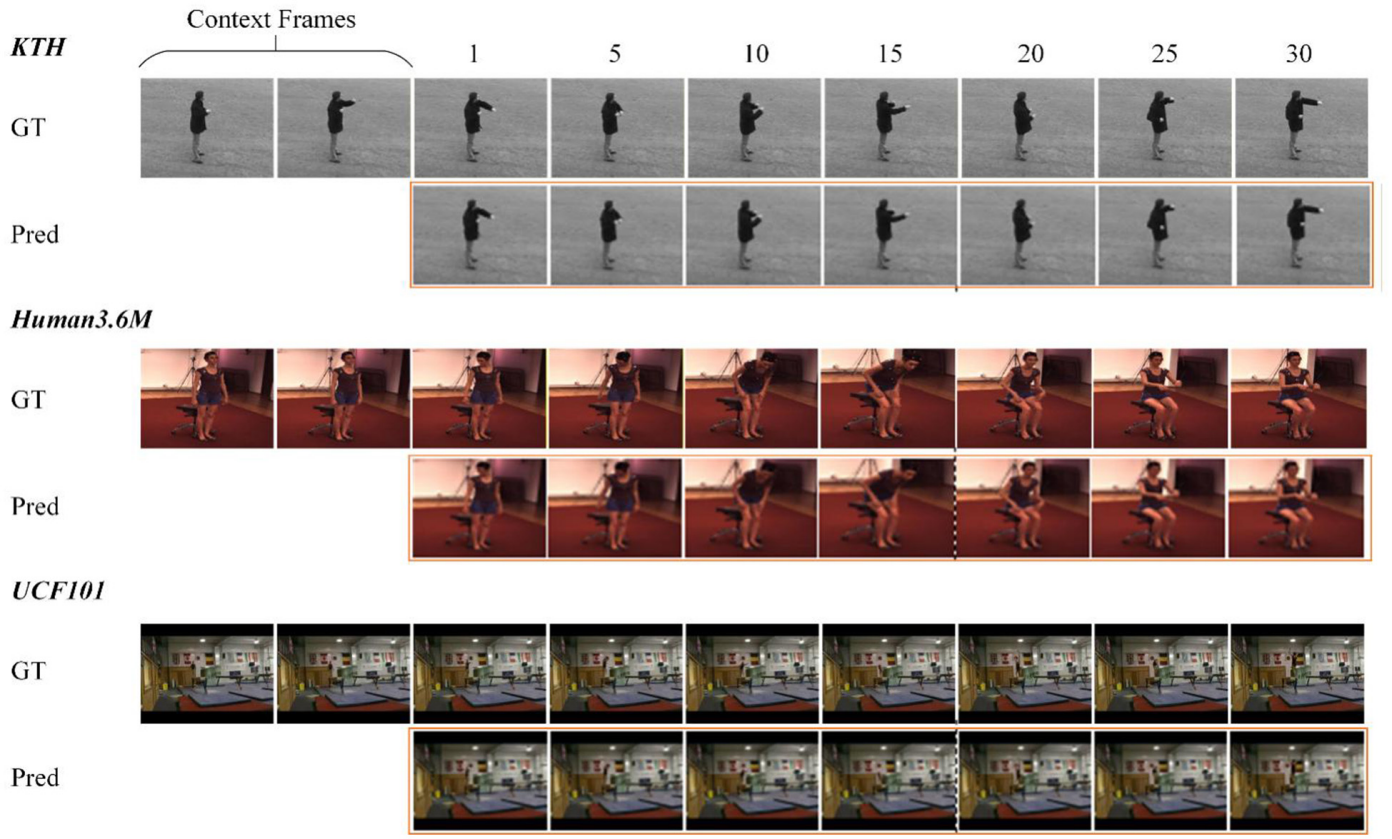
Qualitative visualization of long-range continuous visual anticipation across benchmark datasets. The figure displays sample predictions on KTH **(top)**, Human3.6M **(middle)**, and UCF101 **(bottom)**. For each sequence, the top row shows the Ground Truth (GT) frames, while the bottom row presents the corresponding future frames generated by NeuralVisionNet (Pred). The model is conditioned on the initial context frames to anticipate the dynamics for the subsequent 30 frames.

Table 1 presents the quantitative evaluation of NeuralVisionNet against state-of-the-art stochastic baselines, including an ablation variant NeuralVisionNet (w/o event gist) that removes the global event representation. Our framework demonstrates superior performance across all datasets, particularly in metrics reflecting perceptual realism. Figure 2 visualizes the generated sequences across all three benchmarks.

**Table 1:** Quantitative comparison of long-term video prediction (10 → 30 frames) on KTH, Human3.6M, and UCF101 benchmarks.

**KTH**	**k**	**#pred**	**FVD↓**	**PSNR↑**	**SSIM↑**
MCVD (Voleti et al., 2022)	10	30	298	27.8	0.841
RIVER (Davtyan et al., 2023)	10	30	182	29.8	0.850
NeuralVisionNet (w/o event gist)	10	30	174	29.6	0.849
NeuralVisionNet (ours)	10	30	**162**	**30.0**	**0.855**
**Human 3.6M**	
DVG (Shrivastava and Shrivastava, 2021)	10	30	475.2	18.2	0.687
SRVP (Franceschi et al., 2020)	10	30	418.7	19.1	0.710
NeuralVisionNet (w/o event gist)	10	30	235.6	21.4	0.782
NeuralVisionNet (ours)	10	30	**181.4**	**22.8**	**0.817**
**UCF 101**	
MCVD (Voleti et al., 2022)	10	30	537.1	11.7	0.547
RaMViD (Höppe et al., 2022)	10	30	516.2	**14.3**	0.437
NeuralVisionNet (w/o event gist)	10	30	462.5	12.6	0.575
NeuralVisionNet (ours)	10	30	**418.3**	13.2	**0.610**

The proposed NeuralVisionNet is compared against state-of-the-art stochastic baselines including MCVD, RIVER, DVP, SRVP, and RaMViD. Arrows (↑\↓) indicate whether higher or lower values denote better performance. Bold indicates the best result.

Structured Motion (KTH). On datasets with fundamental actions, NeuralVisionNet achieves the best scores across all metrics. Notably, we reduce the FVD to 162, an improvement of approximately 11% over the strong baseline RIVER (182). This confirms that our continuous spatiotemporal representation effectively captures motion physics with fewer artifacts than discrete alternatives.

Complex Human Dynamics (Human3.6M). The bio-inspired architecture shows its most significant advantage here. We achieve an FVD of 181.4, less than half the error rate of the second-best model, SRVP (418.7). To assess the contribution of the global event-gist latent, we compare NeuralVisionNet with an ablated variant that removes this component (NeuralVisionNet w/o event gist). On Human3.6M, the full model improves PSNR/SSIM from 21.4/0.782 to 22.8/0.817 and reduces FVD from 235.6 to 181.4, suggesting that the event-gist latent helps maintain coherent long-horizon dynamics and better preserves human-body structure under complex motion.

Unconstrained Scenes (UCF101). NeuralVisionNet continues to lead in structural modeling, achieving the best FVD (418.3) and SSIM (0.610). While RaMViD shows slightly higher PSNR (14.3 vs., 13.2), its significantly worse FVD (516.2) reflects the well-known limitation where PSNR favors blurry, averaged predictions. In contrast, our model prioritizes perceptual realism and structural sharpness, aligning better with the biological preference for coherent structures over raw pixel accuracy.

## 5 Discussion

### 5.1 Neuro-computational validation of continuous anticipation

Our results provide compelling empirical support for the hypothesis that aligning deep generative models with neuro-computational principles enhances the fidelity of visual anticipation. The superior performance of NeuralVisionNet, particularly in the structure-sensitive FVD and SSIM metrics across KTH and Human 3.6M datasets, suggests that the brain's strategy of separating “event gist” (global latent variables) from specific spatiotemporal instantiations is computationally advantageous. By minimizing the variational free energy (ELBO) via the Attentive Neural Process, our model effectively filters out sensory noise to capture the underlying causal dynamics of the scene. This corroborates the theoretical framework of predictive coding (Friston, 2010), which posits that the visual cortex does not merely process incoming stimuli but actively constructs top-down generative models to explain sensory causes. The significant margin over deterministic baselines highlights that modeling the intrinsic uncertainty of the future-rather than suppressing it-is crucial for generating coherent, non-blurry predictions over long horizons.

### 5.2 The role of grid-like coding in temporal consistency

A key innovation of our work is the integration of grid-like codes to function as a continuous spatiotemporal coordinate system. The experimental success in modeling complex human dynamics (Human 3.6M) validates the functional utility of grid cells beyond spatial navigation, extending their role to “mental time travel” (Bellmund et al., 2018). Unlike discrete Transformers such as Sora or ViViT, which quantize time into fixed tokens (Brooks et al., 2024), our continuous representation allows for the seamless interpolation of motion. This mirrors the entorhinal cortex's ability to represent space and time as a continuous metric, enabling the model to maintain structural rigidity even when extrapolating 30 frames into the future. This finding aligns with recent “NeuroAI” perspectives (Whittington et al., 2020), suggesting that structural generalization in artificial agents relies on separating sensory content (“what”) from structural coordinates (“where” and “when”).

### 5.3 Structural coherence vs. pixel-wise accuracy

An important observation from our evaluation on the UCF 101 dataset is the trade-off between PSNR and perceptual metrics (SSIM/FVD). While NeuralVisionNet achieved superior FVD and SSIM scores, its PSNR was slightly lower than that of the RaMViD baseline. This discrepancy is a well-known phenomenon in stochastic video generation (Mathieu et al., 2015). Deterministic or pixel-focused models often minimize mean squared error by averaging all possible futures, resulting in “safe” but blurry predictions that artificially inflate PSNR. In contrast, our probabilistic approach samples a single, distinct “event gist” from the latent prior, generating a sharp, detailed future that may slightly deviate pixel-wise from the ground truth but preserves high structural realism. From a neuroscience perspective, this suggests that the biological visual system prioritizes the coherence of high-level semantic structures (scene layout, object integrity) over low-level pixel precision, a priority that is successfully reflected in our model's optimization landscape.

## 6 Conclusion

In this paper, we presented NeuralVisionNet, a bio-inspired probabilistic framework that bridges the gap between discrete deep learning and continuous biological anticipation. By synergizing the hierarchical feature extraction of Video Swin Transformers with the stochastic reasoning of Attentive Neural Processes, we introduced a novel grid-like coding scheme that enables “mental time travel” across continuous spatiotemporal coordinates. Our empirical evaluations on KTH, Human 3.6M, and UCF 101 demonstrate that this approach significantly outperforms state-of-the-art baselines in structural consistency (SSIM) and perceptual realism (FVD), validating the hypothesis that separating static visual content from dynamic spatiotemporal representations is essential for long-term prediction. Ultimately, this work offers a computational proof-of-concept for predictive coding theories, suggesting that future artificial visual systems should move beyond fixed-frame discretization toward continuous, probabilistic, and constructive generative processes.

## Data Availability

The raw data supporting the conclusions of this article will be made available by the authors, without undue reservation.
